# RETRACTED: Dua et al. Myricitrin, a Glycosyloxyflavone in *Myrica esculenta* Bark Ameliorates Diabetic Nephropathy via Improving Glycemic Status, Reducing Oxidative Stress, and Suppressing Inflammation. *Molecules* 2021, *26*, 258

**DOI:** 10.3390/molecules30193918

**Published:** 2025-09-29

**Authors:** Tarun K. Dua, Swarnalata Joardar, Pratik Chakraborty, Shovonlal Bhowmick, Achintya Saha, Vincenzo De Feo, Saikat Dewanjee

**Affiliations:** 1 Advanced Pharmacognosy Research Laboratory, Department of Pharmaceutical Technology, Jadavpur University, Kolkata 700032, India; 2 Department of Pharmaceutical Technology, University of North Bengal, Darjeeling 734013, India; 3 Department of Chemical Technology, University of Calcutta, Kolkata 700009, India; 4 Department of Pharmacy, University of Salerno, 84084 Fisciano, Italy

The journal retracts the article “Myricitrin, a Glycosyloxyflavone in *Myrica esculenta* Bark Ameliorates Diabetic Nephropathy via Improving Glycemic Status, Reducing Oxidative Stress, and Suppressing Inflammation” [1] cited above.

Following publication, concerns were brought to the attention of the Editorial Office regarding the integrity of a number of images presented in this publication [1].

Adhering to our standard procedure, an investigation was conducted by the Editorial Office and Editorial Board that identified indications of inappropriate editing and partial duplication between figures presented in this article [1] and other publications [2,3], produced by a different research group and presenting different experimental conditions. While the authors collaborated within this process, raw material meeting the journal’s requirements for original images in *Molecules* could not be provided for Editorial Board evaluation. Consequently, the Editorial Board has lost confidence in the reliability of the findings and has decided to retract this publication, as per MDPI’s retraction policy (https://www.mdpi.com/ethics#_bookmark30, accessed on 14 July 2025).

This retraction was approved by the Editor-in-Chief of the journal *Molecules*.

The authors did not agree to this retraction.
